# Effectiveness and safety of omega-3 fatty acids for the prevention of ischemic complications following carotid artery stenting: An early terminated pilot study

**Published:** 2018-01-05

**Authors:** Farzaneh Foroughinia, Elaheh Jamshidi, Haniyeh Javanmardi, Anahid Safari, Afshin Borhani-Haghighi

**Affiliations:** 1 Clinical Neurology Research Center, Shiraz University of Medical Sciences, Shiraz, Iran; 2 Department of Clinical Pharmacy, School of Pharmacy, Shiraz University of Medical Sciences, Shiraz, Iran; 3 Student Research Committee, Shiraz University of Medical Sciences, Shiraz, Iran; 4 Stem Cells Technology Research Center, Shiraz University of Medical Sciences, Shiraz, Iran; 5 Department of Neurology, School of Medicine, Shiraz University of Medical Sciences, Shiraz, Iran

**Keywords:** Stroke, Intracranial Hemorrhages, Carotid Stenosis, Angioplasty, Stent, Omega-3 Fatty Acids, Platelet Aggregation Inhibitors

## Abstract

**Background:** We aimed to study the possible beneficial effects of omega-3 polyunsaturated fatty acids (PUFAs) in carotid artery stenting (CAS) procedure for decreasing post-procedural ischemic complications. Although previous evidence demonstrated that omega-3 PUFAs, present in fish oil, can significantly enhance platelet response to antiplatelet agents after percutaneous coronary intervention (PCI), it is unknown whether they can be used in patients undergoing CAS.

**Methods:** The single-blind, case-control, pilot randomized trial study was planned to perform on 60 patients undergoing CAS (30 in case and 30 in control group). Patients in both groups were pretreated with dual antiplatelet therapy (aspirin 80 mg/day with a loading dose of 325 mg, and clopidogrel 75 mg/day after a loading dose of 600 mg) at least 48 hours before the CAS. 30 patients randomly received 3000 mg loading dose of omega-3 fatty acids 12 hours before the procedure and 1000 mg omega-3 capsule the day after the procedure. All subjects were planned to be visited by neurologist for any peri- and post-procedural complications immediately after the procedure and on first, seventh, and thirtieth days.

**Results:** We ended the study after the enrollment of 18 patients because of an unexpected hemorrhagic transformation in case group. Two patients in this group developed hemorrhagic symptoms less than 12 hours after the procedure. One of the failures occurred in a patient with small vessel disease. Except these two cases, no one showed any neurological deficit symptoms in both groups.

**Conclusion:** In patients already receiving dual antiplatelet treatment before CAS, adding omega-3 PUFAs would increase the incidence of hemorrhagic transformation.

## Introduction

Stroke is one of the most common causes of death and disability all over the world.^1^ Large arterial disease including carotid artery stenosis is one of the significant but treatable causes of ischemic stroke.^2^ Carotid endarterectomy (CEA) and carotid angioplasty and stenting (CAS) are main therapeutic options for carotid stenosis.^3^ Transient ischemic attack, ischemic strokes, intracerebral hemorrhage (ICH), myocardial infarction (MI), hemodynamic depression, and death are the most serious complications of CAS.^3^^,^^4^

Administration of dual antiplatelet therapy with aspirin and clopidogrel before and after CAS is routinely recommended for prevention of ischemic complications. Individuals taking aspirin and clopidogrel may have resistance to these antiplatelet drugs and may remain at higher risk of ischemic complications.^5^ In a recent clinical trial study by Foroughinia, et al., it was shown that omega-3 ingestion can significantly decrease the risk of myonecrosis and infarction among patients undergoing percutaneous coronary intervention (PCI) without any major complication.^6^

Omega-3 polyunsaturated fatty acids (PUFAs) [eicosapentaenoic acid (EPA) and docosahexaenoic acid (DHA)] are the important components of cellular membrane.^7^ These essential fatty acids are naturally derived from fish oil. DHA and EPA can inhibit platelet aggregation^8^ and have antithrombotic and anticoagulant properties as well.^9^^,^^10^ They also act as neuroprotective agent and can significantly improve the outcomes of brain ischemia by their anti-inflammatory properties.^11^^,^^12^

Regarding to the prevalence and importance of post-procedural ischemic complications of CAS and dual antiplatelet therapy failure in some patients, and the positive effects of omega-3 PUFAs in preventing infarction after PCI in previous studies, we decided to evaluate the effect of omega-3 fatty acids consumption on post-CAS complications in patients already premedicated with dual antiplatelet therapy.

## Materials and Methods

This single-blind randomized clinical trial (RCT) was conducted in September to December 2016 at Kowsar hospital, Shiraz University of Medical Sciences, Iran. Patients were examined by stroke neurologists and subsequently referred to the research principal investigator for recruitment. 

Consecutive patients with ischemic stroke confirmed via a brain computed tomography (CT) scan, or magnetic resonance imaging (MRI) were recruited. 

Noninvasive vascular studies including Doppler ultrasound of cervical arteries, transcranial Doppler sonography of intracranial arteries, CT angiography and/or MR angiography were performed in appropriate patients. Cardiac evaluations and laboratory tests were performed for all patients. 

Patients with intracranial hemorrhage (ICH), cardio-aortic embolic strokes, lacunar infarcts, and non-atherosclerotic causes of carotid stenosis were excluded. Patients with Modified Rankin Scale (MRS) of equal or greater than 3, and patients who had contraindications for angiography were excluded, too. 

The percentage of stenosis was calculated based on the North American Symptomatic Carotid Endarterectomy (NASCET) criteria.^13^ Symptomatic patients with stenosis of 50% or more, and asymptomatic patients with stenosis of 70% or more in angiography were included. Both high risk and standard risk patients for CEA were recruited. 

At the time of registration, we recorded current or previous cigarette smoking state, hyperlipidemia, hypertension, and the history of diabetes mellitus according to the international criteria previously mentioned in our previous publications.^3^

To best of our knowledge, this was the first randomized clinical trial to compare standard pre-stenting dual antiplatelet therapy and dual antiplatelet plus omega-3; so, sample size calculation was impossible. Accordingly, a pilot study was conducted. We arbitrarily planned to randomize successive patients undergoing CAS to two groups of case and control with 30 patients in each. The randomization sequence was computer generated and the statistician was blinded to which group received omega-3 supplements.

During CAS, 80 units heparin per kilogram of body weight to maintain an activated clotting time (ACT) time of 250 seconds or more were administered. Distal embolic protection devices were used for all patients. Then, a self-expanding stent was placed across the narrowed segment of carotid arteries. Pre- and/or post-dilation were performed in suitable cases.

Both case and control groups were pretreated with standard medication regimen at least 48 hours before the procedure. Standard medications include clopidogrel 75 mg daily after a loading dose of 600 mg, and maintenance dose of 80 mg of aspirin daily with a loading dose of 325 mg. Case group patients also received 3000 mg (three softgel capsules) loading dose of omega-3 fatty acids 12 hours before the procedure. Each omega-3 capsule contained 330 mg EPA and 220 mg DHA (Premium V Life Company, UK). Both groups were supposed to receive clopidogrel 75 mg per day and aspirin 80 mg per day at least for one year after the procedure. 

Patients were supported over the next days and were planned to be examined by neurologist for ischemic or hemorrhagic stroke and any other peri- and post-procedural complications at predetermined intervals (immediately after the procedure and on the days 1, 7, and 30). 

Primary outcome was composite outcome of any stroke, MI and/or mortality. Secondary outcomes were ischemic stroke, ICH, MI, and death.

We used SPSS software (version 16, SPSS Inc., Chicago, IL, USA) for data analysis. The continuous values presented as means ± standard deviation (SD), and categorical variables presented as counts and percentage. 

This study was approved by the institutional review board (IRB) of Shiraz University of Medical Sciences (RCT code: IRCT2017011120441N6), and all the participants gave written informed consent after they had been informed about the study.

## Results

A total of 60 patients (30 cases and 30 controls) who planned to undergo CAS, were recruited in this study. The trial was terminated after the enrollment of 18 patients due to an unexpected hemorrhagic stroke after decoding both hemorrhagic strokes was found to be in case group.

***Patient Characteristics:*** Table 1 shows the preoperative, intraoperative, and postoperative characteristics of case and control groups. Table 2 shows the preoperative, intraoperative, and postoperative characteristics of patients in case group with or without hemorrhagic transformation. There were 4 women and 4 men, ranging in ages from 65 to 82 years (mean 75.2 ± 6.2) in case group, and 5 women and 5 men in control group. The mean age in control group was 71.7 ± 6.8 years (range: 57-83). No statistically significant difference was present between the two groups (P > 0.05). 

***Angiographic and stenting results:*** Stenting procedure was performed in 6 right and 2 left carotid arteries in case group and 3 right and 7 left carotid arteries in control group. No one had bilateral carotid artery stenting. The mean stenosis of the operated carotid artery in case and control groups were 75.8% and 78.9%, respectively. Mean contralateral carotid artery stenosis percentage was 49.8% in case, and 52.0% in control group. And the mean residual stenosis were 30.0% in case, and 24.0% in control patients. Closed-cell stents were used in all subjects in both groups. The technical success rate in both group were 100%.

**Table 1:** Demographic and baseline disease characteristics of all patients

**Variable**	**Case group (n = 8)**	**Control group (n = 10)**
Men/Women (n)	4/4	5/5
Age (year) (mean ± SD)	75.2 ± 6.2	71.7 ± 6.8
Diabetes mellitus (n)	4	3
Hypertension (n)	8	8
Hyperlipidemia (n)	5	7
Smoking (n)	3	2
Symptomatic target vessel (Right/Left) (n)	6/2	3/7
Mean degree of stenosis (%)		
Target carotid artery	75.8	78.9
Contralateral carotid artery	49.8	52.0
Symptomatic (n)	5	4
Stent type (Open cell/Closed cell) (n)	0/8	0/10
Embolic protection device (n)	7	8
Pre-dilation (n)	3	4
Post-dilation (n)	5	8
Residual stenosis (%)	30.0	24.0
Intraprocedural hypotension/ hypertension (n)	8/0	9/0
Post-procedural ischemic stroke (Non-disabling/Disabling) (n)	0/0	1/0
Post-procedural hemorrhagic stroke (n)	2	0
Post-procedural hypotension/hypertension (n)	8/0	10/0
Extracranial hemorrhage (n)	1	5

SD: Standard deviation

**Table 2:** Demographic and baseline disease characteristics of patients in case group with or without hemorrhagic transformation

**Variable**	**With hemorrhagic ** **transformation**	**Without hemorrhagic ** **transformation**
Men/Women (n)	1/1	3/3
Mean age (year)	77.5	75.16
Diabetes mellitus (n)	1	3
Hypertension (n)	2	6
Hyperlipidemia (n)	1	4
Smoking (n)	1	3
Symptomatic target vessel (Right/Left) (n)	2/0	4/2
Mean degree of stenosis (%)		
Target carotid artery	79.5	74.6
Contralateral carotid artery	74.5	41.6
Symptomatic (n)	1	4
Stent type (Open cell/Closed cell) (n)	0/2	0/6
Embolic protection device (n)	2	5
Pre-dilation (n)	1	2
Post-dilation (n)	2	3
Residual stenosis (%)	25.0	31.7
Intraprocedural hypotension/hypertension (n)	2	5
Post-procedural ischemic stroke (Non-disabling/Disabling) (n)	0/0	0/0
Post-procedural hemorrhagic stroke (n)	2	0
Post-procedural hypotension/hypertension (n)	2/0	6/0
Extracranial hemorrhage (n)	0	1

In case group, 2 patients had unilateral stenosis and bilateral carotid stenosis was present in 6 of them. Severe stenosis (more than 90%) was found in 3 individuals. The procedure was well tolerated by all without significant complications except in two patients. These cases (briefly reported bellow) developed hemorrhagic transformation less than 12 hours after the procedure. One of the failures occurred in a patient with small vessel disease. Except these two patients, no one showed any neurological deficit symptoms in this group and discharged a day after the procedure by a neurologist. 

***Case with hemorrhagic transformation***

*Patient 1: *An 82-year-old man underwent left carotid artery stenting for critical stenosis (about 99% narrowing according to NASCET criteria). He had 40% stenosis in right internal carotid artery (RICA) bulb. He had a recent stroke with right sided weakness and mild aphasia a few weeks before admission. But, as his MRS was 2, it was decided that CAS should be done for him.

**Figure 1:**
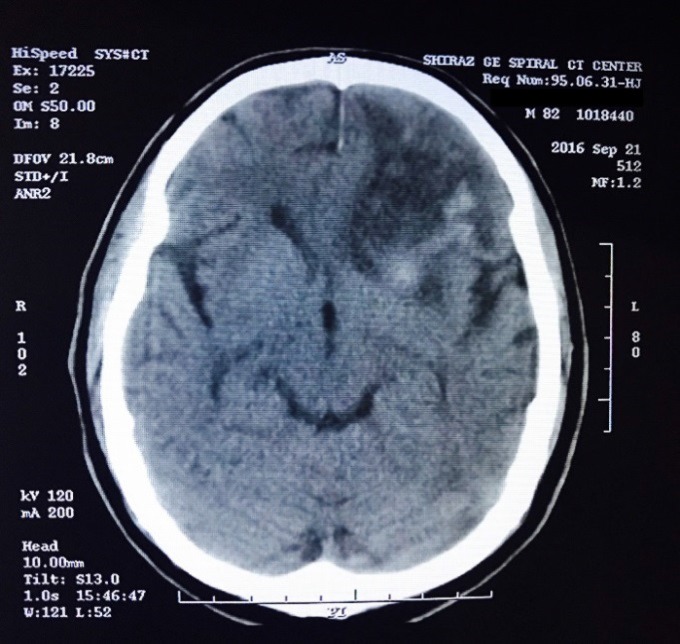
Brain computerized tomography (CT) scan indicating a hemorrhage in the left frontoparietal lobe

Stenting was well tolerated by the patient. Postoperative angiogram revealed a 30% residual stenosis. His postoperative blood pressure was 160/90 mmHg. He remained well until 8 hours after stenting procedure, when he developed headache and aggravation of right-side weakness. CT and MRI demonstrated a rim of hemorrhagic transformation at the periphery of old stroke lesion (Figure 1). Aspirin, clopidogrel, and omega-3 stopped and conservative treatment was advised. The patient improved in a few days and clopidogrel, and aspirin was restarted after 5 and 7 days, respectively.

**Figure 2:**
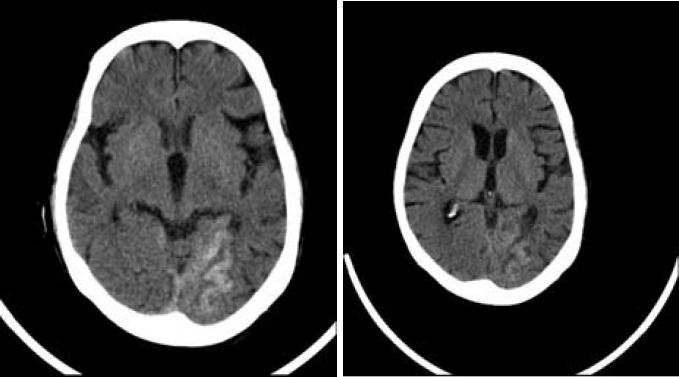
Computerized tomography (CT) scan showing a hemorrhage in the right occipital lobe

*Patient 2:* A 73-year-old woman, with positive history of smoking, hypertension, hyperlipidemia, and type 2 diabetes mellitus underwent stenting for symptomatic RICA stenosis. She had a recent attack with right sided weakness and paresthesia and right homonymous hemianopia. Brain CT and MRI showed territorial infarction in territory of left posterior cerebral artery. Pre-procedural cerebral vessels angiography showed asymptomatic 70% stenosis of proximal part of RICA. She tolerated the procedure well, and the RICA stenosis resolved (residual stenosis < 20%). Postoperative blood pressure was not more than 166/90 mmHg. Intravenous nitroglycerin was infused until the blood pressure was stabilized. Twelve hours after the stenting procedure, the patient presented with convulsion, severe delusions, and urinary incontinence. Hematological and biochemical laboratory data were within the normal range. Brain CT showed gyral hemorrhage in bilateral occipital areas (Figure 2). Aspirin, clopidogrel, and omega-3 stopped and conservative treatment was advised. The patient improved gradually and hemorrhage was disappeared in successive CTs. Clopidogrel and aspirin was restarted after 7 and 12 days, respectively.

## Discussion

This is a report of a pilot study for evaluating the potential advantage of adding omega-3 supplements to aspirin and clopidogrel therapy before CAS. As we confronted two patients with ICH in omega-3 users, we stopped this study. The odds ratio for hemorrhagic stroke were raised significantly in omega-3 users compared with the control group. Despite this adverse effect, there were no major ischemic stroke in case patients at 30-days follow-up. In addition, the risk of minor ischemic strokes was lower in patients who got omega-3 (37% vs. 50%) in small group of case and controls. The result of a trial by Foroughinia, et al. indicates that omega-3 PUFAs administration can successfully reduce the risk of cardiovascular events (MI and necrosis) after PCI without any major complications.^6^ In that study, omega-3 PUFAs decreased the cardiac biomarker creatine kinase-MB (CK-MB) and troponin I in patients with acute coronary syndromes undergoing PCI. Contrary to PCI, adding omega-3 PUFAs to standard antiplatelet treatment in current study did not provide favorable outcome in CAS.

Omega-3 fatty acids (EPA and DHA) have potential to reduce the incidence of thrombus formation by decreasing the platelet aggregation,^14^^-^^16^ therefore reducing the major complications in patients undergoing CAS.

Hemorrhagic complications in dual antiplatelet therapy reported in many studies.^17^^-^^20^ In a recent study, based on the Nationwide Inpatient Sample (NIS) in the United States, acute ICH occurred in 0.15% of patients undergoing CAS.^21^ The rate of 30-day hemorrhagic complications in patients pretreated aspirin and clopidogrel before CAS was 0% and 0.67% in Iranian^3^ and American^22^ studies, respectively. The different outcomes of adding omega-3 PUFAs to aspirin and clopidogrel in PCI and CAS might be justified by the differences between the microvasculature of the brain and myocardium. Coronary and brain microvascular endothelial cells are different according to cytokines, signal transduction enzymes, groups of growth-related molecules, metabolic enzymes, and stress-related proteins.^8^

Cerebral hyperperfusion syndrome may explain hemorrhage particularly in patients who underwent CAS with near total stenosis. Of 450 patients undergoing CAS in Abou-Chebl, et al. study, cerebral hyperperfusion syndrome occurred in 5 cases with hypertension (1.1%). All of these 5 patients had severe stenosis (˃ 90%) before the stenting. They showed that prolonged hypoperfusion may induce arteriolar dilation and impair autoregulation, causing lack of reconstruction ability.^22^

The second patient with hemorrhagic transformation in this study, had definite radiologic evidence of small vessel disease of brain. Hypertension, smoking, and diabetes are major risk factors for small vessel disease.^23^ Deep ICH is positively associated with the cerebral white matter lesions and deep brain infarcts.^24^ Small vessel disease is also associated with the rapid development of cerebral microbleeding after acute ischemic stroke.^25^ Accordingly, the presence of small vessel disease might be considered as a hazardous factor of bleeding in the patients who received omega-3 fatty acids in addition to dual antiplatelets.

In current study, the control group did not receive placebo. This should be considered as a major limitation for the current study.

## Conclusion

As an early-terminated pilot study, current study showed the importance of evolution of ICH in the outcome of patients undergoing CAS. For decreasing the rate of ischemic complications after CAS, other policies rather than adding omega-3 PUFAs should be investigated.

## Notes:

**How to cite this article:** Foroughinia F, Jamshidi E, Javanmardi H, Safari A, Borhani-Haghighi A. **Effectiveness and safety of omega-3 fatty acids for the prevention of ischemic complications following carotid artery stenting: An early terminated pilot study**. Iran J Neurol 2018; 17(1): 11-7.
